# Living Conditions Influence Psychological Distress of Migrants in Long-Term Imprisonment

**DOI:** 10.3389/fpsyt.2019.00818

**Published:** 2019-11-07

**Authors:** Maximilian Lutz, Judith Streb, Manuela Dudeck

**Affiliations:** Department of Forensic Psychiatry and Psychotherapy, Ulm University, Ulm, Germany

**Keywords:** prisoners, migrants, psychological stress, living conditions, European Union

## Abstract

**Background:** Serving a long-term prison sentence places a heavy psychological burden on inmates. The concept of salutogenesis and the psychological stress model developed by Lazarus indicate that people can handle difficult situations if they are able to use their resources in a way that makes them feel confident that things will work out as well as can reasonably be expected. However, during long-term imprisonment inmates often have restricted access to potential coping strategies, such as close and trusting relationships. Because of migration-related difficulties, such as poor local language skills and experiences of discrimination, migrants in long-term imprisonment probably experience even more psychological distress than native citizens.

**Aims:** The aim of the study was to compare the amount of psychological distress in migrants and native citizens in long-term imprisonment. In addition, we investigated whether any aspects of living conditions in prison reduce psychological distress.

**Methods:** From the 1,101 participants in the European Union (EU) project “Long-term imprisonment and the issue of human rights in member states of the EU,” we chose 49 migrants, defined as people born in a different country from where they were imprisoned, and 49 native citizens matched for prison, age (+/–5 years), and index offense. The participants completed a questionnaire that included the Brief Symptom Inventory (BSI) and 128 items from a revised version of the Mare-Balticum prison survey. Data were analyzed by multilevel regression models.

**Results:** Native citizens reported higher psychological distress than migrants. However, multilevel regression analyses showed that poor relationships with fellow inmates and increased fear of crime were significant predictors of increased psychological distress in migrants only.

**Conclusions:** Being a migrant by itself does not lead to increased psychological distress in prisoners. This finding can be explained by the so-called healthy immigrant effect. However, migrants experience psychological distress when prisons are not safe and when they do not have close and trusting relationships with fellow inmates.

## Introduction

In Europe, imprisonment “consists only of the extensive curtailment of the freedom of movement” (1). According to the European prison rules, imprisonment “shall not aggravate the suffering inherent in prison” (2). Nevertheless, long-term prisoners in the European Union (EU) have severe psychological symptoms. The descriptive analysis of psychological symptoms within the EU project “long-term imprisonment and human rights” indicates that 57.7% to 86.1% of inmates in prisons in Europe require treatment for psychological disorders (1). The worldwide prevalence of severe psychological disorders in male prisoners is 3.6% for psychosis and 10.2% for depression (3), indicating that psychological distress is a widespread problem during imprisonment. A 2016 review questioned whether mental illness is imported into prison or whether imprisonment itself causes mental illness and found evidence that after imprisonment symptoms of depression decreased whereas psychotic symptoms remained stable (4). Suicidality was found to increase during imprisonment if prisoners experience conditions such as overcrowding and violence or are in higher security prisons (5). Taken Together, findings suggest that institutional conditions may at least partly explain psychological distress in prison.

How do some prisoners manage to stay healthy while others do not? The concept of salutogenesis implies that people tend to stay healthy under highly stressful conditions if they have access to resources such as ego identity, social support, continuance, and cultural stability that they can use to get “a dynamic feeling of confidence that one’s internal and external environments are predictable and that there is a high probability that things will work out as well as can reasonably be expected” (6, 7). In accordance with Lazarus’ theory of cognitive appraisal, healthy people tend to classify threatening situations as manageable (8).

Long-term imprisonment in particular represents a heavy psychological burden. Besides being deprived of liberty, prisoners are deprived of autonomy, heterosexual relationships, security, and personal possessions for a barely manageable length of time (4). Because prisoners have access to few resources, such as close and trusting relationships, coping successfully may be quite difficult. The situation may be even worse for subgroups such as migrants. Several studies have shown that factors associated with migration (e.g., language difficulties, separation from close relatives, uncertain residence status) make migrants in general particularly vulnerable to mental distress or mental illness. Migrants report lower psychological well-being than native citizens (9–11) and are more likely to have depression, psychosomatic complaints, posttraumatic stress, substance abuse, and increased suicidality (12, 13). However, the relationship between migration and psychological distress remains unclear because other studies found contradicting results. According to the so-called healthy immigrant effect, migrants in general are healthier and more resilient than the nonmigrant population/their national peers in the host country because only those people leave their home country who can withstand the strain of migration. Thus, most prime-aged migrants are positively selected, they are more educated and in better psychological and physical healths than are nonmigrants. This effect has been documented among migrants in Europe (14, 15), the United States (16, 17), and Canada (18, 19).

Furthermore, bicultural identity has been found to have mental health benefits. When migrants are devalued by the receiving society, identification with the heritage culture increases, which ensures supportive relationships within the ethnic community. These relationships buffer the negative impact of perceived discrimination on well-being. This effect only occurs, however, when there is a high ethnic density in the community (20). In summary, the effects of migration on distress depend on individual characteristics and social context.

On the basis of the above findings, the present study aimed to compare the amount of psychological distress in migrants and native citizens in long-term imprisonment in an explorative manner. We hypothesized that migrants would have a higher amount of psychological distress because they have fewer social resources and may feel more isolated. In addition, we wanted to investigate whether any aspects of living conditions in prison can reduce psychological distress.

## Methods

### Sample and Procedure

The data were collected for an EU-wide study on long-term imprisonment and human rights. Prisons that house long-term prisoners were identified by project partners in the participating countries. All eligible prisoners were informed that there would be a survey about their everyday life and well-being and were asked to participate. Thus, the sample consists of all those who volunteered. The researchers met participants in small groups and were able to help participants with literacy problems. Participants participated voluntarily and were not promised any kind of incentive. The groups of prisoners varied in size depending on the rooms. The study was carried out in accordance with the Declaration of Helsinki. The study had 1101 participants and was conducted at 36 institutions in 11 European countries between 2007 und 2009. Descriptions of the whole sample and project can be found in Drenkhahn et al. (1). The participants completed a paper-and-pencil questionnaire that included the Brief Symptom Inventory (BSI) and 128 items from a revised version of the Mare-Balticum prison survey. The study defined long-term imprisonment as a sentence of at least 5 years.

For the current analysis, we chose a matched sample of male migrant-native pairs. Inmates were classified as migrants if their country of birth was different from their country of imprisonment. We identified a total of 90 migrants and then matched them with native prisoners on the basis of prison (exact match), age (with a tolerance of +/–5 years), and index offense (exact match). We were able to match 49 migrants and 49 native citizens, resulting in a total of 98 participants. In addition to matching, it was examined whether the two groups, migrants and natives, differ with regard to other influential variables (length of accommodation, educational level and variables for social support). **Table 1** shows that the two groups did not vary significantly.

**Table 1:** Descriptive statistics.

	Migrants *n* (%)/*M* (*SD*)	Native Citizens *n* (%)/*M* (*SD*)	Statistics
Age (in years)	38.18 (9.41)	37.86 (9.33)	*t*(96) = –.173, *p* = .863
Index offense			(parallelized)
*Homicide*	27 (55%)	27 (55%)	
*Robbery*	4 (8%)	4 (8%)	
*Sexual offense*	4 (8%)	4 (8%)	
*Assault*	1 (2%)	1 (2%)	
*Theft/Fraud*	3 (6%)	3 (6%)	
*Drug offense*	10 (20%)	10 (20%)	
Country of detention			(parallelized)
*Germany*	4 (8%)	4 (8%)	
*Croatia*	5 (10%)	5 (10%)	
*France*	3 (6%)	3 (6%)	
*Lithuania*	5 (10%)	5 (10%)	
*Denmark*	5 (10%)	5 (10%)	
*England*	10 (20%)	10 (20%)	
*Sweden*	6 (12%)	6 (12%)	
*Belgium*	2 (4%)	2 (4%)	
*Finland*	4 (8%)	4 (8%)	
*Spain*	5 (10%)	5 (10%)	
Lengths of imprisonment (in months)	75.49 (56.80)	83.65 (60.02)	*t*(93) = .68, *p* = .498
Lengths of imprisonment to be served			*X*²(3) = 1.40, *p* = .706
*Unlimited*	13 (28%)	13 (28%)	
*Limited: first third*	6 (13%)	7 (15%)	
*Limited: second third*	20 (43%)	16 (34%)	
*Limited: last third*	7 (15%)	11 (23%)	
Education			*X*²(2) = 3.10, *p* = .245
*University/College degree*	2 (4%)	6 (12%)	
*Graduated school*	45 (92%)	39 (80%)	
*No graduation*	2 (4%)	4 (8%)	
Being married or in a partnership	20 (41%)	12 (25%)	*X*²(1) = 2.74, *p* = .131
Receiving visits from family members or friends	36 (73%)	35 (71%)	*X*²(1) = .97, *p* = .483

Data were analyzed with IBM SPSS Statistics for Windows Version 25 (Armonk, NY: IBM Corp.). The analysis comprised three steps: First, we computed the intraclass correlation coefficient (ICC) within prisons; the ICC was 26.19%, indicating that we should use multilevel analysis to control for related errors within prisons. Second, we estimated differences in the amount of psychological distress between migrants and native citizens with multilevel regression models. Third, we used multilevel regression models to predict the amount of psychological distress, with the various aspects of prison conditions and migration status as independent variables. To estimate these parameters, we used the maximum likelihood method.

### Brief Symptom Inventory

The Brief Symptom Inventory (BSI, 11) is a self-report measure of psychological distress. It consists of 53 items (α = .97) divided into nine subscales, i.e., anxiety, depression, interpersonal sensitivity, hostility, obsessive-compulsive behavior, psychoticism, paranoid ideation, phobic anxiety, and somatization (21). Items are rated on a 5-point Likert scale ranging from 0 (not at all) to 4 (extremely) and summed to a total score. Higher scores indicate a higher amount of psychological distress. Total scores are transformed into T-values, which are normalized according to sex and age.

### Long-Term Imprisonment Survey

The long-term imprisonment survey was a revised version of the Mare-Balticum prison survey, which was first used in the Mare-Balticum prison study (22). The survey has 128 items that capture various domains, including accommodation, health, work and education, free time, contacts within the institution and with the outside world, security, problems, and conflicts. For the present study, we analyzed the 10 scales listed below; on each of the scales, higher scores indicated a higher degree on the respective dimension. An item analysis of the present data found a Cronbach’s α of *r* = .71 to *r* = .87.

1. *Cell comfort* (maximum score = 11) summed up how well the prison cell was equipped. It contained dichotomous items that asked whether equipment such as a toilet was available and whether climatic conditions were adequate.
2. *Cell stressors* (α = .83, 6-point Likert scale) asked about the amount of distress related to noise, air, temperature, light, lack of privacy and personal items, and fellow prisoners.
3. *Value of work* (α = .77, 4-point Likert scale) comprised items that captured the subjective meaning of work in prison.
4. *Activity time* summed up how many hours per week a prisoner did something such as working, exercising, or other regular activities.
5. *Relationships with prisoners* (α = .72, 4-point Likert scale) comprised items that asked to what extent relationships with other prisoners were supportive and respectful.
6. *Relationships with ward staff* (α = .84, 4-point Likert scale) comprised items that asked to what extent relationships with ward staff were supportive and respectful.
7. *Fear of crime* (α = .87, 4-point Likert scale) comprised items that asked to what extent prisoners were afraid of becoming a victim of blackmail, theft, humiliation, sexual abuse, or rape.
8. *Experience of crime* (binary variable: yes = 1/no = 0) asked whether the prisoner had been blackmailed, robbed, humiliated, sexually abused, or raped inside the prison.
9. *Frequency of conflicts* (α = .71, 4-point Likert scale) comprised items that captured the frequency of getting into conflict with prison rules, ward staff, or prisoners.
10. *Contact frequency outside prison* (α = .86, 6-point Likert scale) comprised items that asked how often prisoners were in touch with people outside the prison, including visits, phone calls, and letters.

## Results

### Differences in Psychological Distress Between Migrants and Native Citizens

Psychological distress was higher in native citizens (marginal mean = 48.32, 95% CI = [45.27, 51.37]) than in migrants (marginal mean = 44.75, 95% CI = [41.70, 47.80]). This difference (3.57, 95% CI = [.48, 6.67]) was significant (*t* (75.59) = 2.30, *p* = .024).

The group of migrants includes migrants from countries within the EU (*n* = 12) and migrants from countries outside the EU (*n* = 37). It was examined whether these two subgroups differ in their mean BSCL values (*M* *_intra EU_* = 44.17, *SD* *_intraEU_* = 10.17, *M_extra EU_* = 45.03, *SD* *_extraEU_* = 9.46). A *t*-test does not become significant (*t*(47 = .269, *p* = .789).

### Moderating Effects of Migration in Regressions Between Prison Conditions and Psychological Distress

**Table 2** shows the correlation coefficients between prison conditions and psychological distress, and **Table 3** shows the means and standard deviations of all scales for native citizens and migrants. Pairwise comparisons found that native citizens reported more cell stressors and experienced more crime than migrants. The results of the linear mixed models predicting psychological distress can be seen in **Table 4**. The result of four of the nine analyses were significant, and cell stressors and experience of crime were significant predictors. Prisoners who reported multiple cell stressors and who experienced crime had greater psychological distress. The interaction migration x relationships with prisoners was significant. Good relationships with fellow prisoners were a protective factor against psychological distress only in migrants. The interaction migration x fear of crime was significant, indicating that migrants who did not feel safe and who were afraid of assaults reported an increased level of distress.

**Table 2:** Correlations between psychological distress [Brief Symptom Inventory (BSI)-total] and prison conditions in a sample of migrants (*n* = 49) and native citizens (*n* = 49) in long-term imprisonment in 10 European countries.

		1	2	3	4	5	6	7	8	9	10	11
1	BSI-total	1										
2	Cell comfort	–.26^**^	1									
3	Cell stressors	.46^**^	–.44^**^	1								
4	Value of work	.06	.20	–.17	1							
5	Activity time	.04	.12	–.03	.04	1						
6	Relationships: prisoners	–.31^**^	.25^*^	–.15	–.01	.16	1					
7	Relationships: ward staff	–.21^*^	.29^**^	–.33^**^	.26	.20	.33^**^	1				
8	Fear of crime	.39^**^	–.25^*^	.22^*^	.02	–.16	–.45^**^	–.22^*^	1			
9	Experience of crime	.31^**^	–.17	.14	–.23	–.07	–.28^**^	–.37^**^	.41^**^	1		
10	Frequency of conflicts	.03	–.14	.06	.04	–.08	–.01	–.41^**^	.09	.31^**^	1	
11	Contact to outside	–.18	.27^*^	–.15	–.07	–.01	.26^*^	–.02	–.09	–.04	–.04	1

* = *p* < 0.05, ** = *p* < 0.01.

**Table 3:** Means and standard deviations of each prison condition in a sampled of migrants (*n* = 49) and native citizens (*n* = 49) in long-term imprisonment in 10 European countries.

	All Participants		Migrants		Native Citizens		
	*M*	*SD*	*M*	*SD*	*M*	*SD*	*t*
Cell comfort	7.06	1.88	7.10	1.79	7.02	1.98	–.22
Cell stressors	3.74	1.33	3.45	1.36	4.03	1.25	2.19*
Value of work	3.16	.69	3.17	.73	3.14	.66	–.12
Activity time	46.06	24.95	44.38	23.99	47.63	25.99	.60
Relationships: prisoners	3.16	.58	3.27	.57	3.06	.58	–1.79
Relationships: ward staff	2.70	.69	2.81	.64	2.60	.72	–1.51
Fear of crime	1.64	.66	1.63	.67	1.65	.67	.10
Experience of crime	.58	.50	.47	.50	.69	.47	2.15*
Frequency of conflicts	1.70	.78	1.72	.78	1.67	.78	–.28
Contact to outside	4.39	1.14	4.45	1.08	4.33	1.21	–.51

*p < .05, t-tests were computed between native citizens and migrants.

**Table 4:** Linear mixed models predicting psychological distress associated with prison conditions and migration status (b = estimates of fixed effects. random effect: prison) in a sample of migrants (n = 49) and native citizens (n = 49) in long-term imprisonment in 10 European countries.

	*b*	95 % CI *(b)*
**Cell comfort**		
Cell comfort	-.97	-2.21,.27
Migration * Cell comfort	-.49	-2.21, 1.22
**Cell stressors**		
Cell stressors	2.31*	.51, 4.10
Migration * Cell stressors	.47	-1.94, 2.89
**Value of work**		
Value of work	.52	-4.51, 5.54
Migration * Value of work	-.19	-6.47, 6.51
**Activity time**		
Activity time	.01	-.08, .11
Migration * Activity time	-.08	-.22, .06
**Relationships: prisoners**		
Relationships: prisoners	-1.29	-5.43, 2.85
Migration * Relationships: prisoners	-6.16*	-11.91, -.40
**Relationships: ward staff**		
Relationships: ward staff	-3.12	-6.38, .15
Migration * Relationships: ward staff	-.90	-5.95, 4.14
**Experience of crime**		
Experience of crime	8.20**	2.77, 13.63
Migration * Experience of crime	-3.93	-11.34, 3.47
**Fear of crime**		
Fear of crime	1.29	-2.32, 4.91
Migration * Fear of crime	7.23**	2.32, 12.27
**Frequency of conflicts**		
Frequency of conflicts	.93	-2.56, 4.42
Migration * Frequency of conflicts	-1.54	-6.38, 3.29
**Contact to outside**		
Contact to outside	.01	-2.23, 2.20
Migration * Contact to outside	-2.21	-5.34, .91

* p < .05, ** p < .01, b = unstandardized regression coefficient, CI =Confidence intervall, Dependent variable: T-scores of BSI-total. Main effect “migration” was included in the model but is not displayed. Variable “migration”: 1 = migrant, 0 = native citizen.

## Discussion

In this study, we were reanalyzing data of the EU-wide study on long-term imprisonment and human rights in an explorative manner. We hypothesized that migrants in long-term imprisonment would have a higher amount of psychological distress than native citizens because they have fewer social resources and experience more isolation. However, both the null hypothesis significance testing and confidence intervals showed the opposite result, i.e., less psychological distress in migrants. Thus, we conclude that being a migrant may be not associated with higher psychological distress in long-term imprisonment. This finding is in line with studies that found that first-generation migrants have better mental and physical health than native citizens, a phenomenon known as the healthy immigrant effect, as described above (20, 23). However, typical explanations of the healthy immigrant effect do not explain our data. One explanation of the healthy migrant effect is that there is some kind of self-selection effect in that younger, better educated and therefore healthier people are more likely to migrate (23). In the present study, an age-based selection effect can be excluded because of matching. Education-based selection effects are difficult to evaluate because educational qualifications are difficult to compare between EU countries. However, our analysis of the level of education did not find any meaningful difference between migrants and native citizens. Another explanation of the healthy immigrant effect is that supportive relationships within an immigrant’s ethnic community can buffer the negative impact of migration that may arise from discrimination (20). The participants in our study lacked access to their own ethnic community because of imprisonment, however, so that such buffering effects also cannot explain the lower psychological distress of the migrants. Therefore, although the present data support the healthy immigrant effect, none of the explanations outlined above apply.

In addition, our study revealed that migration moderates the relationship between psychological distress and the quality of relationships with fellow inmates. We observed that bad relationships with fellow inmates were associated with increased psychological distress in migrants but not in native citizens. This finding is in line with studies showing that loneliness is associated with current and longitudinal depressive symptoms (24) and with studies showing that social support protects against psychiatric symptoms (25). Thus, the coping factor *relationships with prisoners* appears to be more important for migrants than for natives. One can speculate that the effect may be related to experiences of isolation because migrants belong to a minority group.

Furthermore, our data revealed that for migrants the fear of crime was a significant predictor of psychological distress. Interestingly, migrants stated that they were less likely than native citizens to be victims of crimes in prison, such as being blackmailed, robbed, humiliated, sexually abused, or raped. Thus, even though migrants experienced less crime, they had a greater fear of it. One possible explanation might be that migrants take violence personally and interpret it as a kind of discrimination whereas native citizens attribute conflicts with others to their rough living conditions in prison.

In our study, migration moderated the association between psychological distress and both fear of crime and quality of relationships with fellow inmates. The moderation of both these associations suggests that the scales predicted psychological distress only in migrants. Considering the strong negative correlation between the quality of relationships and fear of crime (r = –.45), one could speculate that these scales measured different aspects of a common factor, e.g., social resources. Experiences of crime may be more likely to be interpreted as personal when they happen under conditions of few social resources. Such experiences of perceived social isolation may lead to prisoners being afraid of fellow inmates and therefore increase their psychological distress.

The results further revealed that native citizens feel more affected by cell stressors than migrants. *Cell stressors* were defined as the inmates’ subjective evaluation of distress caused by their living conditions. The present study also asked about the objective stressors of the immediate environment, which was represented by the variable *cell comfort*. However, cell comfort was rated similarly by migrants and native citizens. Therefore, differences in cell stressors are rather due to differences in appraisal than in objective conditions in prison cells. If we consider the higher amount of psychological distress in native citizens, the results are in line with the vulnerability hypothesis, which states that a high amount of psychological distress increases vulnerability. According to the model of Lazarus (8), imprisonment-related stressors are more likely to lead to a negative appraisal when a prisoner’s general amount of psychological distress is high.

We also examined those factors that were not significant predictors of psychological distress, i.e., *value of work* and *activity time*. The general strain theory proposed by Robert Agnew states that prison environments can lead to a high amount of distress because imprisonment hinders positively valued goal orientation (26). Working and leisure activities should enable prisoners to achieve positively valued goals, experience self-efficacy, and escape from negative stimuli. Generally speaking, working and leisure activities should be resources to cope with psychological distress. However, our data did not show such positive effects. One explanation may be that both activity time per week and value of work are rather associated with the concept of well-being than with psychological distress. A 2012 study compared these constructs and showed that they are related but not similar. It found that items that are positively related to well-being are often negatively related to psychological distress and vice versa (27). Hence, correlations between both constructs are about r = –.35 (28). In addition, studies on leisure time found that the quantity of leisure time is not as important as the quality (29). Thus, the missing effect of activity time per week may be because we measured psychological distress and not well-being or the quantity of leisure time and not the quality.

Although good relationships with inmates were important, contacts to the outside did not predict psychological distress in either migrants or native citizens. This result is in line with a recent study that found that a lack of family support (e.g., not being loved or valued) did not predict mental illness in inmates during their time in prison and only did so after their release (30).

## Limitations

One important limitation concerns the proportion of migrants in this study, which used data from the EU project “Long-term imprisonment and the issue of human rights in member states of the European Union.” A total of 1,101 prisoners participated, and 8.2% met the criterion of being a migrant. According to the official annual penal statistics of the Council of Europe, a mean proportion of 18.01% migrants was registered in those countries that participated in the EU project in 2009 (31). Thus, the proportion of 8.2% migrants in the present study was comparatively low and we must assume that there was a selection bias. There are two possible reasons for this selection error. First, all participants received a questionnaire in the language of the host country, so that the questionnaire could only be answered by the migrants who had sufficient language skills. Second, in many European countries, criminals may lose their right of residence as a result of the conviction and are sent back to their home countries. This happens in particular when they have to serve long-term prison sentences, like the prisoners in the present study.

In the present study, we reanalyzed data from the EU long-term imprisonment project to get insight into migration-related differences in psychological distress. Because the EU project was not designed to study migration-related effects, the following simplifications were necessary: First, we had to choose a criterion that enabled us to define a prisoner as a migrant; we favored birth country over nationality because it showed higher correlations with native language and is more precise in identifying first-generation migrants. However, using the definition “born in another country” as a proxy measure for being a migrant could lead to misclassification. For example, a German child whose parents were living in Geneva at the time of his birth but returned to Berlin when he was 2 months old and lived there for 40 years would presumably be classified as a migrant. The child of Eritrean refugees who fled their homeland when it was in utero but was born in Sweden (a second generation migrant) would not. Thus, one should consider, that migrants as defined by this study are probably a hugely heterogeneous group. Second, psychological distress was predicted on the basis of the available scales, and third, concepts that are meaningful in the context of migration (e.g., ethnic density, acculturation) were absent. Third, the data was collected over 10 years ago (between 2007 and 2009). It must be taken into account, that prison populations and immigration has changed considerably in the last decade.

There are limits to generalizability in a study like this because of the small percentage of eligible prisoners from each country who participated. In this sense, the study is exploratory and tentative. The findings need cautious interpretation in the light of national, regional and local particularities. On the other hand, it provides a first systematic comparison of traumatization and distress in European penal systems.

## Conclusions

Being a migrant by itself did not lead to increased psychological distress, but migrants who had poor or missing social relationships with fellow inmates and those who were more afraid of experiencing crime showed significantly increased distress. Prisons should be made aware of these parameters and should create an environment that supports migrants in building social relationships with fellow prisoners. Furthermore, they should be sensitive to the increased safety needs of migrants.

## Data Availability Statement

The raw data supporting the conclusions of this manuscript will be made available by the authors, without undue reservation, to any qualified researcher. Requests to access the datasets should be directed to maximilian.lutz@uni-ulm.de. 

## Ethics Statement

As anonymous data (without codelist or personal reference) were collected in the EU study, advice from an ethics committee was not indicated. The subjects were informed about the course and purpose of the study, participated voluntarily and received no incentive. The study was carried out in accordance with the Declaration of Helsinki.

## Author Contributions

JS and MD conceived the topic of the paper. MD was responsible for the survey of the EU long-term imprisonment study. ML conducted the literature search and statistical analysis and wrote the first draft of the manuscript. JS supervised the statistical analysis. JS and MD supervised the writing process and revised the manuscript.

## Funding

The original project “Long-term imprisonment and the issue of human rights in member states of the European Union” was funded with financial support from the AGIS Programme European Commission—Directorate-General Justice, Freedom, and Security.

## Conflict of Interest

The authors declare that the research was conducted in the absence of any commercial or financial relationships that could be construed as a potential conflict of interest.
